# American Pharmaceutical Association—Annual Address of President Saunders

**Published:** 1878-12

**Authors:** 


Medical and Surgical Journal.

Vol. XVI.] DECEMBER—1878. [No. 9

### Original Communications.

### AMERICAN PHARMACEUTICAL ASSOCIATION—ANNUAL ADDRESS OF PRESIDENT SAUNDERS.

*Delivered in Concordia Hall, Atlanta, Nov.* 26, 1878.

In consequence of the general alarm which prevailed in the whole of the United States and Canada on account of the rapid spread of yellow fever in some of the towns and cities along the Mississippi during the months of July and August, it was feared that a successful meeting of our Association could not be held here at the time originally fixed, namely, the first week in September, and hence it was deemed desirable to postpone it until now. That dire scourge having disappeared even in the worst affected districts, the impediments to travel have been removed, commerce is assuming her wanted busy aspect, and we meet once more together under happy auspices. Our annual gatherings are always seasons of enjoyment, but it seems to me that on this occasion there is promise of more than the ususl quota of pleasure in store for us. To many of us, especially to those who live so far north as I do, this Southern country is a new world. We found a pleasing novelty in watching the changing aspects of nature in our journey southward, as many trees and shrubs long familiar, gave place to forms new and strange; and had we been privileged to visit here earlier in the season, before nature had lost her summer charms, our pleasure would have been greatly enhanced in beholding the beautiful plants and flowers which adorn the fields and woods, the feathered songsters which fill the groves with melody, and the brilliant insects which flutter in the sunshine.

Our pleasures on such occasions as these are of a mixed character. We are happy in our work. Loyalty to the interests of the Association, coupled with a strong desire to have it fulfill its mission in the advancement of the best interests of the pharmacist; the promotion of right and the suppression of wrong-doing in all the departments in which we are engaged—this, gentlemen, is a motive which has been the magnet drawing many so far Sonth; and most of us would be happy anywhere, with almost any sort of surroundings, in realizing the accomplishment of these higher purposes. Pharmacists can afford to be upright, truthful and honest in their business, to maintain their integrity, notwithstanding the temptations by which they are beset; and if I know anything of the aims of this Association, they are to make us all better men as well as better pharmacists.

The more strictly scientific aspects of our meeting also delight many hearts. The prospect of acquiring new ideas and new light upon old methods of thought and old processes, around which, perhaps, have clustered difficulties which we hoped to have removed. Besides which, there is the social element which we can all appreciate. The presence of our friends makes us happy. There is such a charm in the smile of old familiar faces, and the hearty shake of hands, and the warmer greetings swelling from the hearts of those whom a year or years have separated, give a thrill of pleasure; and as long as the members of this Association continue to be so thoroughly happy in each other's society, and so emulous of promoting each other's welfare, these social features in our annual gatherings will always secure an attendance. True, we miss familiar faces as one after another of our members is called away from among us; but we are buoyed with the hope of meeting them again in that "beautiful land beyond the river."

The itinerant character of these gatherings offers many advantages to our members. The year before last we were charmed with our entertainment in Philadelphia, when that great Centennial Exhibition lent an interest to our meetings such as we never had before. Last year you were the recipients of the hospitality of your Northern friends in the Dominion of her Majesty in Canada; now we move more than two thousand miles south to meet our Southern and other friends in Georgia. Thus the pharmacist, closely pent up as he usually is, behind his counter or in his office during the greater part of the year, has some good opportunities given him of acquainting himself with the extent and resources of his country, the products of the soil, its geological strata, the character of its fauna and flora, and last but not least, of making friends everywhere.

But I must leave these pleasing generalities, and try and present you with something a little more substantial. It is expected, I believe, of your retiring President, that he should, in the annual address which closes his official labors, endeavor to elucidate or bring together some scientific facts bearing on or related to pharmacy, and I have thought that it might perhaps be profitable for us to look for awhile at the state of pharmacy at the time of the discovery of America, and to note in some detail how the art of medicine has been indebted to the New World for many of its most valued officinal articles.

When, on the 14th day of August, A.D., 1492, Columbus set sail on his ever memorable voyage, he left behind him a very different Europe from that of the present day. The manners of the people were little less than barbarous, and history at this time chiefly records a series of treasons, usurpations, murders and massacres. Even the kings and courts had very narrow and selfish views, seldom looking beyond present advantages. The elegance and purity of the Latin tongue was then the highest and almost the only point of a scholar's ambition. Mathematical learning was little valued or cultivated. The true system of the heavens was not dreamt of; there was no knowledge at all of the real form of the earth, and in general, the ideas of mankind did not extend beyond their sensible horizon. Just about this time there was an extraordinary coincidence of events. Closely associated with the discovery of America was the invention of printing, the improvement of navigation, the revival of ancient learning, the reformation and the discovery of the proper method of preparing gun powder so as to make it effective in warfare: events which conspired to entirely change the face of Europe. Up to this time, and for a long period antecedent, the science of medicine had made but little progress; indeed, scarcely any advancement seemed to have been made from the time of Hippocrates, Galen and Celsus, and the same remark will also apply with equal force to a long period subsequent. The works of Celsus in many respects read more modern than do those of the learned Dr. Riverius, physician to the King of France, who obtained great reputation for his medical skill and writings in the early part of the seventeenth century. Among the different classes of medicines referred to by these authors, take as an illustration, anodynes. We find in Celsus prominence given to opium, concentrated decoction of poppies, castor, myrrh, pepper, henbane and hemlock seeds. In Riverius, we have opium also, but associated with such things as decoction of borage, fumitory and cichory, and much stress laid on accompanying external applications, such as marsh mallow root, cow's milk, the excrement of cows and sheep, the yolk of an egg, a live puppy, etc. There were some curious methods of compounding in those days. One of the chapters in Dr. Riverius' Universal Body of Physic, is headed thus "Of the Decoction of an old Cock," wherein it defines that there are two forms of this decoction in use; one he calls an altering decoction, which he says "is used not seldom in chronical diseases, as hypochondriacal melancholy, asthmas, etc.;" the other a purging decoction. The formula prescribes that the old fowl after being wearied with blows and running before he is killed, is to be dressed and stuffed with a mixture of bruised roots and seeds, and then boiled until the flesh be separated from the bones, when the liquid, after being strained, is ready for use. Fancy one of our more plethoric Pharmacists of the present day with this prescription in hand, having carefully selected a venerable male representative of the Gallus banckhira, proceeds to vigorously pursue the unfortunate creature around a small city backyard on a hot summer day, with stick in hand, dealing careful blows so as to ensure that amount of weariness in the victim so necessary to the virtues of the compound, and then after the proper stuffing, watching the long process of ebulition until the leathery structure becomes tender. One experience of this sort would, we think, reconcile him to the most composite of compounds of the present day.

The list of vegetable medicines at that time was large. In 1710 appeared Salmon's English Herbal, a ponderous quarto of over 1,300 pages, profusely illustrated, in which there are described 752 herbs of English growth, including almost every variety of wild and cultivated plants and shrubs then growing in Britain, and to most of them are ascribed virtues of an astonishing character. As a specimen, take the common garden sage. This is said to be "hot and dry in the third degree, astringent, anodyne, carminative, digestive, discussive, diuretick and traumatick, cephalick, neurotick, stomachick, hysterick, anthritick, emmenagogick, sudorifick, alexipharmick and analeptick." It is said to be "good against a vertigo, lethargy, headache from a cold cause, palsie, convulsions, spitting blood, weakness of the nerves, poison, the bitings of serpents and other venomous creatures, the plague and other malarial and pestilential diseases, catarrhs, rheumatisms, etc.'[2] Some idea may be formed as to what is embraced in the et cetera by reading the succeeding pages, where details of the various preparations of this herb are given and their wonderful properties dwelt on. The preparations are: 1st, The green leaves; 2d, The juice; 3d, The essence; 4th, An infusion in wine or water; 5th, A powder of the leaves; 6th, An oil or ointment; 7th, A cataplasm; 8th, Pills; 9th, A gargarism; 10th, A distilled water; 11th, A spirituous tincture; 12th, An acid tincture; 13th, An oily tincture; 14th, A spirit; 15th, A distilled oil; 16th, The potestates or powers; 17th, An elixir, and 18th, A conserve of the flowers.

The animal remedies of the same period might be reckoned by the hundred, for even as late as 1730 the second edition of the Edinburgh Pharmacopœia contained no less than 84 of them. This second edition claimed to be a great improvement on the first, for in the preface we are told that it differs from the former edition in that "some things are left out as not differing from others in virtue, or as having been introduced by the superstition or credulity of antiquity. In this edition there are included four hundred and seventy-five vegetable remedies, only ninety of which are now officinal, and out of the total number there were but fourteen remedies of American growth. The animal substances in the same work numbered 84, only thirteen of which are now retained, and of these, two are used almost exclusively as perfumes. The mineral preparations numbered fifty-seven. Many tinctures were added in this edition, a class of remedies just then coming into use, and said to be generally acceptable to the patient by the agreeable smallness of their dose. The doses of medicines at that time were very large, a quarter of a pint was a common sized dose, a wineglassful a rather small one. Of tinctures there were twenty-six in all, also six elixirs and two wines—wine of ipecac and wine of millepedes. The method of preparing the latter was as follows: Take 300 live millepedes, bruise them a little, and pour thereon a pint of Rhenish wine; let them infuse for a night and afterwards press out the wine. This the authors remark, "is a commodious way of obtaining the virtues of the millepedes, and thus they may be exhibited to great advantage." Among the preparations there are also 17 distilled waters, to which list, although not distilled, is added frog spawn water, which is prepared by hanging any quantity of frog spawn in a bag and collecting the water which gradually drips from it. There are also 11 compound waters, 3 spirits, 8 infusions, 4 acetums, 12 decoctions, 28 syrups, 5 oxymels, 4 jellies, 5 succus, 2 conserves, 3 lozenges, 16 powders, 15 electuaries, 7 hohocks, 18 pills, 13 troches, 10 oils, 4 balsams, 25 ointments, 20 plasters, 27 distilled oils and 14 extracts.

Late in the seventeenth century Joseph Donatus, a physician of Marseilles, a lover of botany, was sent to America by the French King "to promote botanic knowledge." He brought home with him the seeds of many scarce and curious plants, some of which were said to possess wonderful virtues. In "Pomet's History of Drugs," published in 1701, a list of these articles is printed, numbering one hundred and twenty-one, nearly all of them designated by Indian names, with a short description appended, but of such a character as would puzzle the highest botanical authorities to divine what was meant by them. The following will serve as examples: "Bamatu, with five leaves, a tree that is crooked with a pear leaf and a purple bell-flower. Macenilla, a venemous and milky tree with a sweet fruit like an apple, which the Indians poison arrows with. Mandubi, an American four-leaved plant with a yellow flower. Tobocora, a thorny, venomous sea-tree with a double round leaf and berries turned up with little horns, including in 'em a sort of flat agat like stones."

We can scarcely appreciate the difficulties which workers in all departments of natural sciences labored under before the immortal Linnæus introduced the binominal system of nomenclature. Previous to this a short description, or else some prominent or striking feature in connection with the object referred to, formed a necessary part of the name. For instance, the English lavender, which we now know under the name of lavandula vera, was then referred to as "a small plant which botanists call spica-sive lavandula mas, vel nardus Italica, aut pseudo-nardus, which signifies: Spike, male lavender, Italian or bastard nard. Another example, gentiana lutea was then known to science as gentiana vulgaris major, ellebori albi folio, the larger common gentian with the white hellebore leaf. Burdened with such a cumbrous incubus as this, it is no wonder that natural science made so little progress.

At this early period the business of supplying the public with medicines was divided between two classes of dealers, the druggists and the herbsellers. The former devoted their attention to the drugs from foreign sources and the preparations to be made from these as well as from European herbs, while the latter collected herbs and sold them at wholesale to the druggists for manufacturing purposes, and at retail to the public at large. The character of the general dealers in drugs and chemicals was far below what it is at present. There were no pharmaceutical associations then with the object of disseminating information, and no desire among pharmacists to enlighten their fellow-laborers. On the contrary every little discovery in the way of improvement was guarded with the most zealous secresy for fear that some competitor might share in the advantages which it conferred; indeed, men seemed to have the idea that they had a sort of patent right to the results of every advance they might make in the knowledge of their business and the mysteries of the trade were handed down from father to son with the most scrupulous care as things never intended for the light. In 1758 was published a work by an anonymous author, which must have made some stir among the pharmacists of that period, as it exposed the evil practices of many and made known a number of processes and formulæ hitherto kept secret. It was entitled, "The Elaboratory Laid Open, or the Secrets of Modern Chemistry and Pharmacy Revealed." From the revelations here made it is evident that at this early period the iniquitous practice of adulteration permeated all departments, and that even among chemical substances there was scarcely one of which there was not a true and fictitious article in the market. The wholesale dealers and manufacturers appear to have been very unscrupulous, and to have imposed upon the retailers, who placed confidence in them, in every way they could, while the retail pharmacists, instead of making their own preparations after the standard formulæ, were content to buy everything their customers needed ready made, and it is stated that "nearly the whole of what is sent into the country differs from the regular and orthodox prescription."

Some important American remedies found their way very slowly into use. When the Edinburgh Pharmacopœia of 1730 was published, cinchona bark had then been known for eighty years, and had been in more general use for twenty-four years; yet there was only one preparation of it, and that a simple watery extract. The following short history of the introduction of cinchona or China Chinæ, as it was then called, is given in this work: "This simple is the bark of a certain tree growing in the West Indies and called by the Spaniards, fever tree, on account of its surprising efficacy in the cure of that distemper." After naming some of the localities from which it was obtained, and briefly describing the tree, it states that "Cardinal de Lugo was the first who brought it into France, in 1650, upon which it was then called by his name, but afterwards by the name of Jesuits' powder, because they had the distributing thereof; the Cardinal, who was of their order, having left them a large quantity. Its use was now neglected until the year 1706, when Dr. Talbot again brought it upon the stage in France, and established its reputation by the numerous cures he performed with it. These cases appeared so extraordinary to the King of France, the great Louis XIV, that by a royal reward he procured the doctor to publish the secret." Its use, however, during the long interval mentioned, was not entirely discontinued. In "Salmon's Practical Physic," published in 1602, among twenty-three remedies for ague occurs the following: "A specific against all manner of agues: Take quin qum, or Jesuit's bark, 2 drachms, beat it into a powder just about the time of using it; infuse it in a good draught of claret or other generous wine for the space of two hours, then give the patient both liquor and powder at once." The enormous price at which the bark was at first held, must have restricted its use to the wealthy. Pomet tells us that when Cardinal Lugo brought it first from Peru it was sold weight for weight for the price of gold, equal to four pounds sterling per ounce. There were some curious remedies in use for intermittents about this time. We extract the following from "Dr. Fuller's Body of Prescripts," published in London in 1710. "Remedy for ague: A cataplasm of herbs: Take Venice tupentine, 2 ounces; juice of plantain, 1 ounce and a half; figs, 3; the yellow paring of orange rind, 2 drachms; bole, 2 drachms and a half; soot, half an ounce; pigeon's dung, 1 ounce and a half; large spider webs, 6; black soap, 4 ounces; vinegar enough to beat it up with. To drive an ague tie this about the wrists, so as to make it bear hard upon the pulses two hours before the fit."

The same work recommends a decoction of chamomile flowers with a little cochineal and salt of wormwood, as a specific in intermittents little inferior even to the famed Peruvian bark. Long after this the bark had many opponents, and the contest waxed warm between these and its advocates for many years. In 1747 John Wesley wrote his book of Primitive Physic. In a postscript to the preface of his work, the following appears: "It is because they are not safe, but extremely dangerous, that I have omitted the four Herculean medicines, opium (the bark), steel, and most of the preparations of quicksilver. Herculean indeed—far too strong for common men to grapple with. How many fatal effects have these produced in the hands of no ordinary physicians! The instances are glaring and undeniable."

About the same time, the celebrated Dr. Tissot wrote his work entitled "Advice with Respect to Health," in which he strongly recommends the use of Peruvian bark, especially in agues. An anonymous reviewer of the period thus criticises him on this point: "I refer to his vehement recommendation of Peruvian bark as the only infallible remedy either for mortifications or intermitting fevers. He really seems transported with the theme, as do many physicians besides. It is not an infallible remedy either for one or the other. I have known pounds of it given to stop a mortification, yet the mortification spread till it killed the patient. I myself took pounds of it when I was young, for a common tertian ague, and that after vomiting, yet it did not, would not effect a cure; and I should probably have died of it had I not been cured unawares by drinking largely of lemonade. I will be bold to say, from my personal knowledge, that there are other remedies which more seldom fail. I believe the bark has cured six agues in ten; I know cobweb pills have cured nine in ten. The bark has often stopped mortification, but sometimes it has failed. I object, secondly, that it is far from being a safe remedy. This I affirm in the face of the sun: that it frequently turns an intermitting fever into a consumption. By this means, a few years since, one of the most amiable young women I have known lost her life, and so did one of the healthiest young men in Yorkshire. I could multiply instances, but I need go no further than my own case. In the last ague which I had, the first ounce of bark was, as I expected, thrown off by purging; the second, being mixed with salt of wormwood, stayed in my stomach, and just at the hour the ague should have come began a pain at my shoulder blade. Quickly it shifted its place, began a little under my left breast and there fixed. In less than an hour I had a short cough, and soon after a small fever. From that time the cough, the pain and the fever continued without intermission; and every night, soon after I lay down, came first a dry cough for forty or fifty minutes, then an impetuous one, until something seemed to burst, and for half an hour more I threw up thick fœted pus. Here was expedition! What but a ball could have made quicker dispatch than this infallible medicine? In less than six hours it obstructed, inflamed and ulcerated my lungs, and by this summary process brought me into the third stage of a true pulmonary consumption. Excuse me, therefore, if escaped with the skin of my teeth, I say to all I have any influence over, whenever you have an intermitting fever, look at me and beware of the bark."

Finally, however, the bark conquered the opposing prejudices. About ten years later, we find in a work on the commerce of the European settlements in America, when speaking of this substance, the following remarks: "This medicine, as usual, was held in defiance for a good while by the faculty, but after an obstinate defense they have thought proper at last to surrender. Notwithstanding all the mischiefs at first foreseen in its use, everybody knows that it is at this day innocently and efficaciously prescribed in a great variety of cases; for which reason, it makes a considerable and valuable part of the cargo of the galleons."

Ipecacuanha, a word divided at an early period into two, ipeca and coamna, was another remedy from the new world whose virtues were early recognized, and one which seems to have met, at its introduction, with comparatively little opposition. It was first brought into use in 1677, in France, by Monsieur le Gras, a physician who had made three voyages to America; but it does not appear to have attracted general notice until the year 1700, when "Helvetius, an eminent physician of Holland, brought it into repute by the great number of cures he performed therewith." He was the first person who knew the dose and how to manage it to the best advantage. In one of the early medical works it is recommended in cases of dysentery in the dose of ten grains taken for three successive mornings.

Pareira brava was first brought to France in the year 1706, by the Spanish ambassador, on his return from Portugal. It was then esteemed highly as a lithontriptic, a reputation which it still retains.

In 1856 jalap root, sarsaparilla, sassafras and guaiacum were in general use; and before the end of that century serpentaria, contrayerva, cochineal, balsams of tolu, peru and copaibæ, were similarly established, but it was long before the true properties of all of these were fully discovered, and for many years the most extravagant virtues were ascribed to some of them.

In addition to the articles referred to, America has added the following to our list of materia medica: Podophyllum peltatum, rhus toxicodendron, stillingia sylvatica, geranium maculatum, croton eleuteria, scutellaria lateriflora, gelseminum sempervirens, lobelia inflata, veratrum viride, sanguinaria canadensis, polygala senega, phytolacca decandra, cimicifuga racemosa, hydrastis canadensis, prunus virginiana, xanthoxylum fraxineum, and serpentaria virginica, besides a number of medicinal substances of less note. Many of these found their way very slowly into use. In 1801 Dr. Benjamin Smith Barton, of Philadelphia, published the first part of his "Collections for an Essay towards a Materia Medica for the United States;" the second part, which completes the volume, was issued in 1804. In this work the author enters at some detail into the progress made up to that time in the knowledge of the uses of the remedies indigenous to the United States; and throughout, the attention of the reader is frequently drawn to the fact that the first knowledge we have had of the value of some of the most important of our indigenous remedies has been obtained from the Indians, who, long ere the advent of the white man, used these remedies to combat the diseases to which their race was subject.

Among the remedies to which he calls especial attention are the following: Geranium maculatum and heuchera americana as astringents. Uva ursi was also recommended as an excellent astringent and as a new remedy in nephritis. Although this plant is common to both the old and new world, it does not seem to have attracted much attention until about this time. The black snake root (cimicifuga racemosa) was then classed with astringents, and highly recommended in decoction for putrid sore throat; it was also said to be used by the Indians in rheumatic cases. Wild cherry bark was just being introduced, and was claimed to be useful in intermittents. The bark and flowers of cornus Florida was also said to be valuable in this class of diseases, and had then long been held in high esteem among the Indians in such cases. The medical properties of datura stramonium were but little known, but were being investigated with much care. Seneca snake root is mentioned as a salivative vegetable of which not much was known; it was classed with purgatives, for which property it was most highly esteemed; it was also thought to be diuretic, and Dr. Smith claimed that he had used a strong decoction of the root with success in croup. The emetic properties of sanguinaria canadensis were mentioned, but nothing appears to have been known at this time of the other properties of this valuable root. Asclepias tuberosa, at that time called decumbens, is spoken of as a mild cathartic. Podophyllum peltatum was also used as a purgative in doses of twenty grains, and was said to have some advantages over jalap and rhubarb. The extract of butternut bark was favorably noticed as possessed of purgative powers. The author regrets that this extract is often very carelessly made by the country people, and urges that it should be prepared by the better informed apothecaries, and that this remedy should have a place in the Pharmacopœia of the United States, when such a desideratum shall be supplied. From the same work we learn that at this early period the castor oil plant was cultivated for profit in Maryland, Virginia and Ohio. Lobelia inflata was just then coming into notice, although not much was known of its properties; it was supposed to be a diuretic. Spigelia marylandica was in high favor as an anthelmintic, and was so highly esteemed by the Cherokee Indians that the author says it would sometimes be dangerous for a man to be detected in digging it up to carry it away from the country. The whites learned the anthelmintic properties of this vegetable from the Indians. Hydrastis canadensis is spoken of as a popular remedy in some parts of the United States as a tonic, and was also used externally as a wash in inflammation of the eyes. Xanthoxylum fraxineum was mentioned as a medicine well worthy the attention of physicians, and poke root (phytolacca decandra) as an active medicinal agent, some of the properties of which had long been known; it was said to be valuable especially in chronic rheumatism, and in some cases of scrofula.

In the first edition of the United States Phormacopœia, published in 1820, the number of vegetable remedies is about the same as those now officinal, and a large proportion of them hold the same relative standing now as then. A few things at that time in the primary list have since been placed in the secondary, while several articles then in the secondary list are at present in the front rank with those of primary importance. As a notable example of this I would mention ergot.

Our present officinal materia medica list includes 256 articles, of which Africa supplies twelve—eleven primary and one secondary; Asia, forty-six—forty-one primary and five secondary; Europe, eighty-six—seventy-five primary and eleven secondary, while America furnishes no less than one hundred and twelve in all—sixty-eight of which are in the primary list and forty-four in the secondary.

These vegetable productions are scattered among eighty-one different orders of plants, fourteen of which orders include one hundred and thirty-five remedies, more than one-half of the total number. These fourteen orders, each of which constitutes five or more articles to our materia medica if placed in the order of their relative importance with the proportion contributed by each continent, would read as follows: Compositæ, sixteen—America 4, Europe 11, Asia 2, Africa 1; Leguminosæ sixteen—America 6, Europe 2, Asia 4, Africa 4; Rosaceæ fifteen—America 5, Europe 9, Africa 1; Labiatæ fourteen—America 5, Europe 9; Umbelliferæ ten—America none, Europe 6, Asia 3, Africa 1; Euphorbiaceæ eight—America 5, Asia 3; Ranunculaceæ eight—America 6, Europe 2; Coniferæ eight—America 4, Europe 4; Gentianaceæ six—America 3, Europe 2, Asia 1; Urticaceæ six—America 1, Europe 4, Asia 1; Rutaceæ five—America 3, Europe 1, Africa 1; Liliaceæ five—Europe 2, Africa 3; Granmeæ five—America 1, Europe 4. When we consider the great number of living species of plants now inhabiting the earth, the small proportion of them used as remedies for disease, seems quite insignificant. As an illustration of this, take the flora of the northern United States, including the district east of the Mississippi and north of North Carolina and Tennessee as given in Gray's Manual, and see in those orders which contribute the greatest number of medicinal agents, what proportion they bear to the whole.

In the order of compositæ, we have 276 native species, and of these only four have as yet found their way into our Dispensatory. Of Leguminasæ, which yields six American remedies, we find 95 native species, while Rosaceæ has 56, of which five are recognized as remedies. Labiatæ has 47 American species, only five of which are officinal, and Umbelliferæ 39, none of which have as yet found their way into use. Of Ranunculaceæ, we have 43 natives, six of which are included among our remedies, while Euphorbiaceæ gives five to our materia medica list out of a total of 31 native species. Of Coniferæ, we have 18 species, four of which are classed among our medicinal agents. Gentianaceæ 23, only three of which are in use. Solanaceæ, of which we have 10 American species, supplies us with two remedies; of Urticaceæ, we have 14 natives and only one in use; of Gramineæ 136, only one of which is used; of Liliaceæ, we have 49, not one of which is known as a medicine, while in Rutaceæ, of which we only have three native species, all of them are officinal.

We have thus in these fourteen orders, in the Northern United States alone, no less than 840 species, of which only forty-five are as yet used as remedies, a fraction over five per cent. of the whole; but the number of species we have mentioned gives us only a very partial idea of the richness of the flora of the continent of America, for south and west of the limits embraced in Gray's Manual, there is a wealth of vegetation far exceeding that found in the North; and when we add to these products of the northern portion of the continent the rich and varied flora of South America, our material for experiment far surpasses our knowledge. To avoid misconception, I must mention here that in the forty-five American remedies just referred to are included all those contributed by the whole continent of America, so that, when looked at from this standpoint, instead of amounting to nearly five per cent. in the order enumerated, they probably do not exceed one or two out of every hundred species existing. I would not for a moment advocate the idea that every plant not useful for food must necessarily possess some marked medicinal value, yet I cannot help thinking that amongst this great mass of interested vegetable products, many valuable remedies will yet be found, the discovery of which may mark eras in the progress of the science of medicine.

Dr. Barton, in his valuable work already referred to, well remarks that "the man who discovers one valuable new medicine is a more important benefactor to his species than Alexander, Cæsar, or an hundred other conquerors." Even his glory in the estimation of a truly civilized age will be greater and more lasting than that of these admired ravagers of the world. I will venture to go farther. All the splendid discoveries of Newton are not of so much real utility to the world as the discovery of the Peruvian bark or the powers of opium and mercury in the cure of certain diseases. If the distance of time and the darkness of history did not prevent us from ascertaining who first discovered the properties of the poppy—that sweet, oblivious antidote for alleviating pain and for soothing those rooted sorrows which disturb our happiness; if we could tell who first discovered the mighty strength of mercury, or ascertain who was the first native of Peru that first experienced and revealed to his countrymen the powers of the bark in curing intermittent fevers, would not the civilized nations of the earth, with one accord, concur in erecting durable monuments of granite and of brass to such benefactors of the species? Would not even the savage, who wants not a sense of benefits conferred upon him, be seen to form the tumulus of stones or to raise the green sod, the only monuments his humble condition would admit of his erecting? And may we not yet look for the discovery of medicines as important to mankind as opium, the bark and mercury?

The progress of such discovery will necessarily be slow. To establish fully the claims of any medicinal agent, long series of experiments are necessary—experiments conducted with great care; and even then so much is sometimes credited to the drug which really results from the remedial efforts of nature, that it is difficult to determine what properly belongs to each. While these experiments do not properly come within the sphere in which the pharmacist labors, yet he is often enabled to greatly aid the physician by his knowledge of simples, and of the most effectual methods of preparing them for use.

Did time permit, I might refer in some detail to the immense climatic advantages America offers for the cultivation of many of the most important foreign medicinal agents, and to the interesting fields for experiment yet unexplored. So varied is the climate and soil in different portions of this continent, that with suitable care in the selection of localities, one could scarcely fail in any of these departments. Among the successes, commercially, in this direction, permit me to mention the culture of the castor oil plant in the West, and the supplying to commerce not only enough of this useful article for home consumption, but also an excess for export trade; to the culture of the orange and lemon in Florida and Southern California, and that of the olive in California, and the appearance of Californian olive oil in our markets; also, to the growth, in the same favored State, of the sweet and bitter almond, and the manufacture of the products obtained from these for which we have heretofore been indebted to Europe. As an outgrowth of the extensive culture of the finest varieties of grapes on the Pacific slope, and the manufacture, on a large scale, of excellent wines, we have also the production of considerable quantities of bitartrate of potash, and the valued chemical products of which this is the basis.

But I must not weary you. Enough has been said to place the new world in the front rank among the continents of the earth for the production of the good and the useful and the beautiful, to satisfy the eye and please the tastes, as well as to relieve the distresses of mankind, and enough to show that among the most useful and highly medicinal agents may be ranked some of the products of this beautiful western world.

